# Soft-sensor modeling for l-lysine fermentation process based on hybrid ICS-MLSSVM

**DOI:** 10.1038/s41598-020-68081-4

**Published:** 2020-07-15

**Authors:** Bo Wang, Muhammad Shahzad, Xianglin Zhu, Khalil Ur Rehman, Muhammad Ashfaq, Muhammad Abubakar

**Affiliations:** 1 School of Electrical and Information Engineering, Jiangsu University, Zhenjiang, 212013 Jiangsu China; 2 School of Computer Science and Communication Engineering, Jiangsu University, Zhenjiang, 212013 Jiangsu China

**Keywords:** Biotechnology, Microbiology, Engineering, Mathematics and computing

## Abstract

The l-lysine fermentation process is a complex, nonlinear, dynamic biochemical reaction process with multiple inputs and multiple outputs. There is a complex nonlinear dynamic relationship between each state variable. Some key variables in the fermentation process that directly reflect the quality of the fermentation cannot be measured online in real-time which greatly limits the application of advanced control technology in biochemical processes. This work introduces a hybrid ICS-MLSSVM soft-sensor modeling method to realize the online detection of key biochemical variables (cell concentration, substrate concentration, product concentration) of the l-lysine fermentation process. First of all, a multi-output least squares support vector machine regressor (MLSSVM) model is constructed based on the multi-input and multi-output characteristics of l-lysine fermentation process. Then, important parameters ($\gamma$, $\lambda$, $\sigma$) of MLSSVM model are optimized by using the Improved Cuckoo Search (ICS) optimization algorithm. In the end, the hybrid ICS-MLSSVM soft-sensor model is developed by using optimized model parameter values, and the key biochemical variables of the l-lysine fermentation process are realized online. The simulation results confirm that the proposed regression model can accurately predict the key biochemical variables. Furthermore, the hybrid ICS-MLSSVM soft-sensor model is better than the MLSSVM soft-sensor model based on standard CS (CS-MLSSVM), particle swarm optimization (PSO) algorithm (PSO-MLSSVM) and genetic algorithm (GA-MLSSVM) in prediction accuracy and adaptability.

## Introduction

l-Lysine is the second most leading globally produced amino acids that is being used in animal feeds, pharmaceuticals, cosmetics, food industry and many other daily life applications. The estimated global market is 2.2 million tons which is increasing at 10% rate per year^1^. To meet the increased demand, researchers are looking for alternatives to increase the production instead of increasing the plant capacity which is time consuming and much expensive. One of the best ways to increase the productivity is to monitor and control product concentration (reflecting the most intuitive manifestation of fermentation quality, the higher the product concentration, the better the quality) in real time. An excess amount of accumulation of product in reactor causes catabolic repression or osmotic stress for bacteria^2^. Similarly, cell concentration and substrate concentration are paramount variables to increase the output yield^3,4^. Cell concentration reflects the number of bacterial cells and substrate concentration reflects the growth and reproduction status of the bacteria, which has a close relationship with fermentation metabolism and directly affects the final formation of the product. Measurement of these key variables is necessary to control and optimize the fermentation process in real-time to enhance the productivity. However, it is hard to measure cell concentration, substrate concentration, product concentration during fermentation process in real-time due to the highly time-varying, non-linear and uncertain nature of the fermentation process^5–7^.

Many costly offline analysis methods are often used to measure these key biochemical variables such as dry weight method, direct staining method, optical density method and cell counting method. At the same time, there are some problems, such as large time delay, complex operations, large measurement errors and high infection rate, which cannot meet the requirement of real-time optimization control. Because, these classical methods cannot reflect the current state of the fermentation process in time, and it is difficult to meet the real-time dynamics of l-lysine fermentation process. Soft-sensor technology is introduced to solve these problems^8–10^ which constructs inferential mathematical models that can predict real-time values of unmeasurable variables by making use of those easily measurable variables^11^. The results proved that the soft-sensors technology could effectively improve the process monitoring in real-time and fermentation product quality. The successful implementation of these virtual sensors to a larger extent have revolutionized the fermentation industry.

In soft-sensor modeling of the fermentation process, lab-scale data of inputs (easily measurable in real-time using physical sensors) and outputs (cannot be measured in real-time) is collected offline. A non-linear mapping function between inputs and outputs is constructed using some well known data-driven prediction models. Researchers have proposed many data-driven methods for the soft-sensor technology in fermentation. Liu et al. exploited artificial neural network (ANN) to build a soft-sensor for measuring the key variables of marine alkaline protease MP fermentation process^12^. Chong et al. used support vector machine (SVM) to model penicillin fermentation process and results proved that SVM is better than ANN modelling methods^13^. Sang et al. have proposed a model based on least square SVM (LSSVM) to estimate biomass concentration to facilitate on-line monitoring^14^. As computational time complexity of SVM increases with the increase of the size of the dataset, LSSVM solved the curse of dimensionality limitation and it is less dependent on the size of the dataset which has good generalization ability as compared to radial basis function (RBF) neural network^15,16^.

At present, the traditional LSSVM (multi-input single-output models) has proved its usefulness in many daily life applications but the standard formulation of this algorithm seems unable to efficiently handle multi-output regression problems. In general, it is assumed that outputs are mutually independent, and for each output a new model is constructed individually. As this traditional regression model can only predict a single output, so the useful information about the temporal correlation between different outputs is neglected which results in time consumption and low prediction accuracy. To solve these problems, researchers have designed many multi-output regression algorithms as multi-output least square support vector machine regressor models and proved the effectiveness of multi-output models as compared to single output models^17,18^. In addition, multi-output models are also simple and computationally inexpensive^19^.

Meanwhile, optimization algorithms are employed to optimize the important parameters of data-driven models to increase the prediction accuracy. Chen et al. have used Particle Swarm Optimization (PSO) to optimize the weights and threshold of ANN instead of Back Propagation (BP) because of its inherent problems^20^. Robles et al. presented a method to choose the regularization and kernel parameters of SVM using PSO^21^. Genetic Algorithm (GA) is introduced to optimize SVM parameters^22^. Similarly, many other metaheuristic algorithms like Cuckoo Search (CS)^23^, Ant Colony Optimization (ACO)^24^, and Artificial Bee Colony (ABC)^25^ have been used in many industrial applications.

In this work, a novel multi-output least square support vector (MLSSVM) regressor model is introduced to construct the soft-sensor model of the l-lysine fermentation process. Single output LSSVM model is an improved form of SVM (which overcomes the problem of possible overfitting in ANN), has less time cost and provides efficient generalization ability^26,27^. However, for multi-output problems, the utilization of correlation information among outputs is necessary for accurate modeling. Hence, MLSSVM is employed for multi-output soft-sensor model of l-lysine fermentation which utilizes the correlation information among outputs to find a non-linear mapping function between multi-inputs and multi-outputs. Furthermore, the selection of model parameters of MLSSVM is important for the effective results and prediction accuracy of model. Thus, a good metaheuristic optimization algorithm that has good local and global search ability with fast convergence should be selected to choose the best model parameters. In the process of multi-peak optimization, the CS algorithm has the best performance to obtain optimal solution as compared to the PSO, GA, Differential Evolution (DE) and ABC algorithms^28,29^. However, the local search ability and convergence speed of CS needs improvement because of its fixed values of two parameters, probability ‘$p_a$’ and step size ‘$\alpha$’^30,31^. To overcome these problems and improve the prediction ability, the optimum parameters of MLSSVM are selected by using an Improved Cuckoo Search (ICS) optimization algorithm which has successfully solved the above mentioned problems in CS optimization algorithm and provides optimum parameters of MLSSVM because of it’s improved local and global search ability. The proposed hybrid ICS-MLSSVM regression method is also compared with MLSSVM optimized by standard CS (CS-MLSSVM), PSO (PSO-MLSSVM) and GA (GA-MLSSVM) which shows that ICS-MLSSVM outperforms CS-MLSSVM, PSO-MLSSVM and GA-MLSSVM in terms of prediction accuracy and adaptability. Despite of the fact that, CS, PSO and GA have been very successful in many applications, but every optimization problem has a new unknown search space. According to “no free lunch” (NFL) theorem^32^, an optimization algorithm successful in particular set of optimization problems may not be successful in some other optimization problems. ICS proved to be more competent in our case to avoid local optimal solution and provides best global optimal solution as compared to CS, PSO and GA.

## Methods

### Single-output Least Squares Support Vector Machine (LSSVM)

Suykens et al. proposed LSSVM^33^ by introducing an equality constraint instead of inequality constraint in SVM^34,35^. The convex quadratic programming (QP) problem is converted to a linear system of equations. The basic modeling principle is as follows:

Suppose there are *l* examples for training, $\{(x_i,y_i)|\; i=1,2,\dots ,l\},\; x_i\in R^n$ is an input vector and $y_i \in R$ is corresponding output. LSSVM learns a mapping function between inputs and outputs defined as:

1 $$\begin{aligned} y(x_i)= \omega ^T\varphi (x_i)+b \end{aligned}$$

The optimization problem for regression LS-SVM is as follows:

2 $$\begin{aligned} min\;\;\underset{\omega ,\xi ,b}{J(\omega ,\xi )}&= \frac{1}{2}\omega ^T\omega +\frac{g}{2}\sum _{i=1}^{l}\xi ^2\nonumber \\ \textit{s.t.}\;\;\;\;\;\;y_i(x_i)&= \omega ^T\varphi (x_i)+b+\xi ,\;\;\;\;\;\; i=1,2,\dots ,l \end{aligned}$$

where $\omega$ is a weight vector; $g\in R^+$ is penalty parameter; $\xi _{i}$ is error variable; *b* is deviation; $\varphi (\cdot )$ is mapping to a high dimensional space. Lagrange method is used to optimize the above problems:

3 $$\begin{aligned} \L (\omega ,\xi ,b,\alpha )= \frac{1}{2}\omega ^T\omega +\frac{g}{2}\sum _{i=1}^{l}\xi ^2 -\sum _{i=1}^{l}\alpha _i(\omega ^T\varphi (x_i)+b+\xi _i-y_i) \end{aligned}$$

where $\alpha _i$ is a Lagrange multiplier. According to KKT (Karush–Kuhn–Tucker) conditions, the transformation to the linear equation is as follows:

4 $$\begin{aligned} \begin{aligned} \begin{pmatrix} 0 &{} 1_l^T \\ 1_l &{} \varvec{K}+g^{-1}I_l \end{pmatrix} \begin{pmatrix} b \\ \alpha \end{pmatrix} = \begin{pmatrix} 0 \\ {y} \end{pmatrix} \end{aligned} \end{aligned}$$

where $y=[y_1,y_2,\dots ,y_l]^T$; $1_l=[1,1,\dots ,1]^T$; $I_l$ is $l_{th}$ ordered unit matrix; $\alpha =[\alpha _1,\alpha _2,\dots ,\alpha _l]^T$; $\varvec{K}$ is the kernel function matrix to satisfy Mercer’s conditions:

5 $$\begin{aligned} \varvec{K}=\varphi (x_i)^T\varphi (x_j),\;\;(i,j)=1,2,\dots ,l \end{aligned}$$

This study utilizes RBF kernel function because of its supreme generalization ability and performance^36^;

6 $$\begin{aligned} \varvec{K}=\varvec{K}(x,x_i)=\exp ^\frac{-|x-x_i|^2}{2\sigma ^2} \end{aligned}$$

where $\sigma$ is the kernel function width. Finally, the function of LSSVM is estimated as:

7 $$\begin{aligned} y(x)=\sum _{i=1}^{l}\alpha _i\varvec{K}(x,x_i)+b \end{aligned}$$

### Multi-output Least Squares Support Vector Machine (MLSSVM)

The l-lysine fermentation process is a complex system because the bacteria continue to ingest substances from the external environment into the cells, obtain energy for survival through a series of biochemical reactions, and expel metabolites from the body. During the fermentation process, the growth of bacteria and the formation of products are not parallel. In a specific bioreactor, the relationship between biological growth and process control, environmental impact, reactor characteristics, etc. is intricate. This forms a complex multi-input/multi-output nonlinear system. Considering the nonlinear multiple-input multiple-output (MIMO) characteristics in the fermentation process, the traditional LSSVM method needs to be improved for multi-output problems. At present, the single output regression LSSVM formulation can be easily extended to multiple output MLSSVM. Xu et al. have designed a MLSSVM model to exploit correlation information among outputs^37^. MLSSVM aims at finding a mapping function between multi-input and multi-output space, thus considers the correlation information between different outputs. Given a set of examples $\{(x_i,y_i)|\; i=1,2,\dots ,l\},\; x_i\in R^n$ is an input vector and $y_i \in R^m$ is corresponding output vector. MLSSVM finds a non-linear mapping $R^n \rightarrow R^m$. It find values of $w = (w_1,w_2,\dots ,w_m) \in R^{n_h\times m}$ and $b = (b_1,b_2,\dots ,b_m) \in R^{m}$ by solving the following optimization problem:

8 $$\begin{aligned} min\;\;\underset{w,b}{J(w,\Xi )}&= \frac{1}{2}trace(w^Tw)+\frac{\gamma }{2}trace(\Xi ^T\Xi )\nonumber \\ \textit{s.t.}\;\;\;\;\;\;y&= z^Tw+repmatrix(b^T,l,1)+\Xi \end{aligned}$$

where $\Xi =(\xi _1,\xi _2,\dots ,\xi _m)\in R^{l\times m}$, $z=(\varphi (x_1),\varphi (x_2),\dots ,\varphi (x_m))\in R^{n_h\times l}$, $\varphi : R^n \rightarrow R^{n_h}$ maps to a Hilbert space *H* (feature space), which is higher $n_h$ dimension space, $trace(A)=\sum _{i=1}^{m}A_{(i,i)}$ and *repmatrix*(*A*, *m*, *n*) creates a $(m\times n)$ block matrix tilling copies of a given matrix *A*, ‘$\times$’ denotes a simple multiplication operator. It is assumed that all $w_i\in R^{n_h}$ can be rewritten as $w_i=w_0+v_i$; whereas $(w_0,v_i)\in R^{n_h}$. If the outputs are similar, the vectors $v_i$ are small, and if outputs are different than each other, the mean vectors $w_0$ are small. In other words, $w_0$ bears the information of correlation and $v_i$ bears the contrast information. The objective function for solving $w_0\in R^{n_h}$, $v=(v_1,v_2,\dots ,v_m)\in R^{n_h\times m}$, and $b=(b_1,b_2,\dots ,b_m)\in R^{m}$ is as follows:

9 $$\begin{aligned} min\;\;\underset{w,b}{J(w_0,v,\Xi )}&= \frac{1}{2}(w_0^Tw_0)+\frac{\lambda }{2m}trace(v^Tv)+\frac{\gamma }{2}trace(\Xi ^T\Xi )\nonumber \\ \textit{s.t.}\;\;\;\;\;\;y&= z^Tw+repmatrix(b^T,l,1)+\Xi \end{aligned}$$

where $w=(w_0+v_1,w_0+v_2,\dots ,w_0+v_m,)\in R^{(n_h\times m)}$, $\Xi =(\xi _1,\xi _2,\dots ,\xi _m)\in R^{l\times m}$, $z=(\varphi (x_1),\varphi (x_2),\dots ,\varphi (x_m))\in R^{n_h\times l}$ and $\lambda ,\gamma \in R$ are two real positive regularization penalty parameters. Lagrange method for optimization is as follows:

10 $$\begin{aligned} L(w_0,v,b,\Xi ,A)= J(w_0,v,\Xi )-trace(A^T(z^Tw+repmat(b^T,l,1)+\Xi -y)) \end{aligned}$$

where $A^T=(\alpha _1,\alpha _2,\dots ,\alpha _m)\in R^{l\times m}$ is the Lagrange multiplier matrix. By using KKT condition following set of equations is achieved:

11 $$\begin{aligned} {\left\{ \begin{array}{ll} \frac{\partial L}{\partial w_0}=0 ; \;\Rightarrow v_0=\sum _{i=1}^{m}z\alpha _i,\\ \frac{\partial L}{\partial v}=0 ; \;\;\;\Rightarrow v=\frac{m}{\lambda }zA,\\ \frac{\partial L}{\partial b}=0 ; \;\;\;\Rightarrow A^T1_l=0_l,\\ \frac{\partial L}{\partial \Xi }=0 ; \;\;\;\Rightarrow A=\gamma \Xi ,\\ \frac{\partial L}{\partial A}=0 ; \;\;\;\Rightarrow z^Tw+repmatrix(b^T,l,1)+\Xi -y=0_{l\times m},\\ \end{array}\right. } \end{aligned}$$

From the above equations, it is clear that $w_0$ is a linear combination of $v_i$ as $w_0=\frac{\lambda }{m}\sum _{i=1}^{m}v_i$. As it is assumed that $w_i=w_0+v_i$, so for the above optimization problem, the estimation function in terms of *v* and *b* is rewritten as:

12 $$\begin{aligned} min\;\;\underset{v,b}{J(v,\Xi )}&= \frac{\lambda ^2}{2m^2}(v1_m1_m^Tv^T)+\frac{\lambda }{2m}trace(v^Tv)+\frac{\gamma }{2}trace(\Xi ^T\Xi )\nonumber \\ \textit{s.t.}\;\;\;\;\;\;y&= z^Tv+repmatrix(\frac{\lambda }{m}z^Tv1_m,1,m)+repmatrix(b^T,l,1)+\Xi \end{aligned}$$

In Eq. 8 it can be seen that it tries to find only small size vectors for decoupling between different parameters of outputs. However, in Eq. 12 change in the first term of expression results a problem that finds additionally a tradeoff between small size vectors, $trace(v^Tv)$ and $v1_m1_m^Tv^T$ (closeness to an average vector of all vectors).

A linear system can be achieved similarly to LSSVM by using KKT condition, which yields the following equation of the Linear system:

13 $$\begin{aligned} \begin{aligned} \begin{pmatrix} 0_{ml\times m} &{} P^T \\ P &{} H \end{pmatrix} \begin{pmatrix} b \\ \alpha \end{pmatrix} = \begin{pmatrix} 0_m \\ {y} \end{pmatrix} \end{aligned} \end{aligned}$$

where $P=blockdiag(1_l,1_l,\dots ,1_l)\in R^{ml\times m}$, $blockdiag(x_1,x_2,\dots ,x_l)$ creates a block diagonal matrix with blocks of $x_1,x_2,\dots ,x_l$ at diagonal positions and remaining entities as zero, $1_m=[1,1,\dots ,1]^T\in R^m$, $0_m=[0,0,\dots ,0]^T\in R^m$, $H=\Omega +\gamma ^{-1}I_{ml}+(\frac{m}{\lambda })Q\in R^{ml\times ml}$, $K=z^Tz\in R^{l\times l}$, $Q=blockdiag(K,K,\dots ,K)\in R^{ml\times ml}$, $\Omega =repmat(K,m,m)\in R^{ml\times ml}$, $\alpha =(\alpha _1^T,\alpha _2^T,\dots ,\alpha _m^T)^T\in R^{ml}$, $y=(y_1^T,y_2^T,\dots ,y_m^T)^T\in R^{ml}$. This linear system consists of $((l+1)\times m)$ equations. Finally, the function of MLSSVM is estimated as:

14 $$\begin{aligned} {\left\{ \begin{array}{ll} y(x)&{}=\varphi (x)^Tw^*+b^{*T}\\ &{}=\varphi (x)^Trepmatrix(w_0^*,1,m)+\varphi (x)^Tv^*+b^{*T}\\ &{}=\varphi (x)^Trepmatrix(\sum _{i=1}^{m}z\alpha _i^*,1,m)+\frac{m}{\lambda }\varphi (x)^TzA^*+b^{*T}\\ &{}=repmatrix(\sum _{i=1}^{m}\sum _{j=1}^{l}\alpha _{ij}^*k(x,x_j),1,m)+\frac{m}{\lambda }\sum _{j=1}^{l}\alpha ^{j*}k(x,x_j)+b^{*T} \end{array}\right. } \end{aligned}$$

The linear system in Eq. 13 is hard to solve because the coefficient matrix is not positive definite, so it can be converted to a positive definite linear system by small transformation as follows^37^:

15 $$\begin{aligned} \begin{aligned} \begin{pmatrix} S &{} 0_{ml\times m} \\ 0_{m\times m} &{} H \end{pmatrix} \begin{pmatrix} b \\ H^{-1}Pb+\alpha \end{pmatrix} = \begin{pmatrix} P^TH^{-1}y \\ {y} \end{pmatrix} \end{aligned} \end{aligned}$$

where $S=P^TH^{-1}P\in R^{m\times m}$ and it is a positive definite matrix. The following relation can easily find the solution for $\alpha$ and *b*:

16 $$\begin{aligned} {\left\{ \begin{array}{ll} b&{}=S^{-1}\eta ^Ty\\ \alpha &{}=v-\eta b\\ \end{array}\right. } \end{aligned}$$

where $S=P^T\eta$, $\eta$ and *v* can be calculated from $H\eta =P$ and $Hv=y$; where $H\in R^{ml\times ml}$ is a positive definite matrix.

The selection of the model parameters of MLSSVM (such as penalty factors $\lambda$, $\gamma$ and kernel width control factor $\sigma$) has a critical role in building a soft sensor model. This work employed ICS optimization algorithm to optimize the MLSSVM parameters and replaced the traditional experience and trial-error based methods. The obtained optimal parameters are utilized to build a more accurate soft sensor model.

### Cuckoo search optimization algorithm

Optimization algorithms like GA, PSO and ACO have been proved more successful than conventional algorithms to solve real-world problems. The intuition of CS was taken from the reproduction style of Cuckoo bird that chooses nest of another specie for laying eggs^38^. The host bird may discard an alien egg by identifying it, or abandon the nest to build a nest at a new position by utilizing the Lévy flight idea^39^. The basic rules are: every cuckoo can lay only one egg and randomly selects a nest to hatch it; the optimal solution (best nest) will be preserved for the upcoming generation; there are a fixed number of nests, and the host bird with a chance $p_a\in (0,1)$ may find out the new strange egg. The update in nest position using Lévy flight occurs according to following mathematical relation:

17 $$\begin{aligned} x_i^{(t+1)}=x_i^{(t)}+\alpha \oplus L(\mu ), \quad i=1,2,\dots ,n \end{aligned}$$

where $x_i^{(t)}$, $\alpha$ and *n* refer to the current location of the nest, step size, and the total number of host nests respectively. $\oplus$ stands for entry wise multiplication, $L(\mu )$ suit distributed from Lévy and is a random flight step, where $(1<\mu <3)$. After updating the position, the probability $p_a$ is compared with a randomly generated number $r\in (0,1)$. If $p_a>r$ the nest position remains the same and if $p_a<r$, the nest location $x_i^{(t+1)}$ is changed randomly.

### Improved Cuckoo Search (ICS) optimization algorithm

Although the CS optimization algorithm has excellent global search performance, yet its convergence speed and local search ability still suffers in finding the best optimum solution. The reason is that, the values of ‘$p_a$’ and step size ‘$\alpha$’ are fixed in standard Cuckoo Search algorithm. For larger $\alpha$, the convergence speed decays at a swift rate, which results in the worst performance of the algorithm. For small $\alpha$, the algorithm require too many iterations to reach the best solution. Furthermore, small ‘$p_a$’ increases the quality of the best solution (accuracy) in each generation but solution diversity decreases, whereas with a large value of $p_a$, the solution diversity increases and leads to an immature convergence^40^. In standard CS, ‘$p_a$’ and ‘$\alpha$’ are the key parameters and play an essential part in improving the global and local search ability of the algorithm. Therefore, in this paper, the values of ‘$p_a$’ and ‘$\alpha$’ are selected adaptively. In start the values are adjusted large enough to increase the diversity of solution and with each iteration these values are decreased to increase the fine-tunning ability of solution in later generations. The originally fixed discovery probability is improved according to formula as follows^41^:

18 $$\begin{aligned} p_a(N_i)=p_{amax}-\frac{N_i}{N_{max}} \times (p_{amax}-p_{amin}) \end{aligned}$$

where $p_{amax}$, $p_{amin}$, $N_{max}$ and $N_{i}$ represent the maximum discovery probability, minimum discovery probability, maximum iterations and currently ongoing iteration respectively. The value of step size ‘$\alpha$’ improved according to the following mathematical relation:

19 $$\begin{aligned} \alpha (N_i)&=\alpha _{max} \times exp(k \times N_{i}) \end{aligned}$$

20 $$\begin{aligned} k&=\frac{1}{N_{max}} \times ln\frac{\alpha _{max}}{\alpha _{min}} \end{aligned}$$

### ICS-MLSSVM soft-sensor modeling method

**Figure 1:**
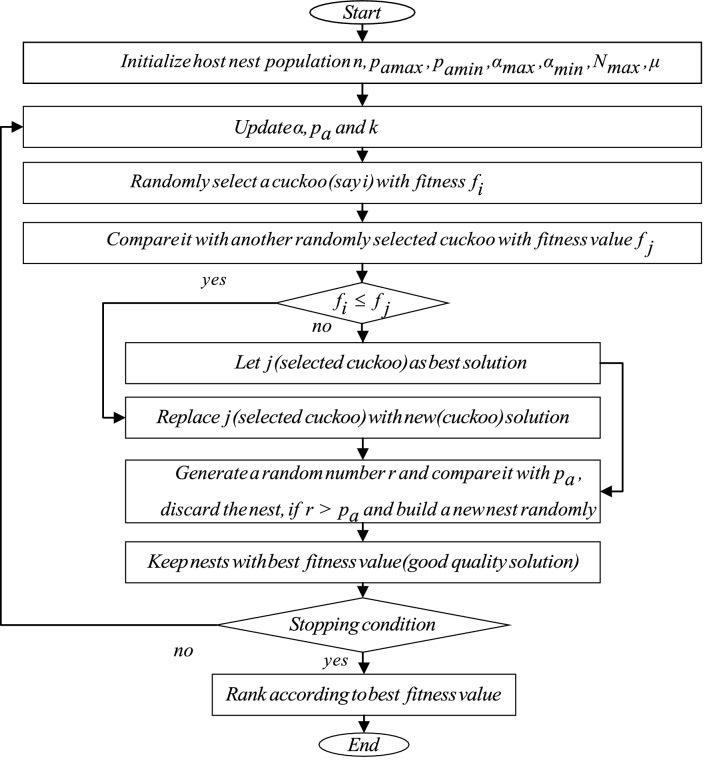
Improved Cuckoo search optimization algorithm.

The model parameters of MLSSVM are penalty factors $\gamma$, $\lambda$ and kernel width control factor $\sigma$, that play an important role in its performance. As in SVM and LSSVM, a very large value of $\gamma$ would lead to remarkably high accuracy on training data but less accuracy on test data, while lower value makes the model less functional and shows poor performance^21^. In addition, an excessively large value of kernel factor $\sigma$ inflicts overfitting problem and small value results under-learning problem. The kernel factor $\sigma$ defines the effect of a single training example on other examples. Therefore, there is a need to choose the values of MLSSVM model parameters $\gamma$, $\lambda$ and $\sigma$ carefully. Researchers have used different optimization algorithms to select the optimum values of critical parameters of regression models. Zhu et al. have used PSO to optimize LSSVM parameters^15^. Based on the above references, we propose to use an “Improved Cuckoo Search” algorithm to find optimum values of MLSSVM parameters. Figure 1, depicts the ICS algorithm in steps. The steps of ICS-MLSSVM are as follows:

Step 1: Prepare train, cross-validation, test dataset and perform normalization.
Step 2: Define $p_{amax}$, $p_{amin}$, $\alpha _{max}$, $\alpha _{min}$, $N_{max}$ (maximum iterations) and generate initial population *n* (total host nests) randomly.
Step 3: Define an objective function. In our work we have used formula 23, where *y*(*i*) and $y'(i)$ are actual and the predicted values respectively. For the current generation, according to the objective function, the optimal solution $f_{min}$ is calculated and reserved for the next generations.
Step 4: For every new iteration, update the values of $p_a$, $\alpha$ and *k* using Eqs. 18, 19 and 20.
Step 5: Randomly select a cuckoo $x_i$ by using Lévy flight with fitness $f(x_i)$ and calculate fitness function at any other randomly selected host nest. If $f(x_i)$ is less than previously stored fitness value, replace that solution (host nest) with a new solution (newly selected nest). Otherwise, leave the same solution for next iterations.
Step 6: Generate a random number $r\in (0,1)$ following uniform distribution and compare it with updated probability $p_a$. If $r>p_a$, randomly change the bird’s nest position and if $r<p_a$, the nest remains unchanged (good quality solution).
Step 7: Test new generation’s nests and keep the best quality solution for the next generation. Finally, the best quality solution $x_b^{(t)}$ (global optimum solution) is selected from a group of bird’s nests with better results and meets the precision requirement $f_{min}$. If it does not fit, repeat from step (4), till $N_{max}$ reaches or meets the precision requirement.

**Figure 2:**
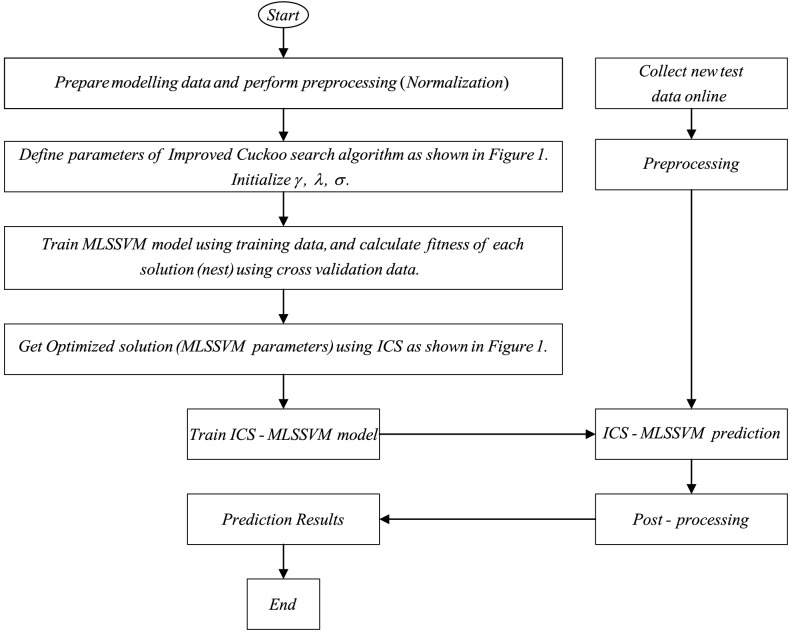
ICS-MLSSVM soft sensor model.

Figure 2 shows the proposed hybrid ICS-MLSSVM soft sensor model.

21 $$\begin{aligned} f=\sum _{i=1}^{n}(y(i)-y'(i))^2 \end{aligned}$$

## Results and discussion

### Experimental setup

The experiment of l-lysine fed-batch fermentation was carried out at the control system platform of Jiangsu University. The RT-100L-Y fermenter model was used to perform this experiment. To make the experiment close to the actual production process, the experimental process was designed as follows:

- The time period for every batch was 72 h and the sampling time period *t* was 15 min. The auxiliary inputs (such as temperature *T*, *pH*, speed of stirring motor *r*, dissolved oxygen $D_o$, acceleration rate of glucose flow $u_1$, acceleration rate of ammonia flow $u_2$ and air flow rate $u_3$) were collected in real-time by testing instrument. The key biochemical variables (cell concentration ‘*X*’, substrate concentration ‘*S*’ and product concentration ‘*P*’) were sampled after every 2-h and tested in laboratory offline. After this, the key biochemical parameters were transformed from 2-h sampled data to 15 min sampled data (consistent with the number of auxiliary inputs data) in *MATLAB* using the “spline” interpolation function *interp1* (https://www.mathworks.com/help/matlab/ref/interp1.html). The cell concentration was achieved after performing some computations using the method of cell dry weight i.e. centrifuge tube was filled with 10 ml liquor of fermentation, the operation of centrifuge was carried out for 5 min at 3000 r/min, supernatant was set aside, and washed by distilled water twice and after that it was dried at $105\;^{\circ }\hbox {C}$ until its weight became constant, then calculated its weight. *S* was measured by SBA-40C multi-function biosensor. *P* was determined by the modified ninhydrin colorimetric method i.e. 2 ml of the supernatant and 4 ml of the ninhydrin reagent were mixed and heated in boiling water for 20 min. The absorbance at 475 mm was measured by a spectrophotometer after cooling and obtained by checking the standard l-lysine curve.
- 10 batches were used for testing the modeling competence of the hybrid ICS-MLSSVM method. The initial conditions between batches were set differently and the feeding strategy was also changed to enhance the differences between batches. The pressure of the fermentation tank was set to 0.1 MPa, the temperature of fermentation was adjusted at $30\;^{\circ } \hbox {C}\pm 10\;^{\circ } \hbox {C}$ and the dissolved oxygen electrode was calibrated for the reference reading when the stirring motor was rotating at 400 r/min.

### Evaluation metrics

In addition, to assess the prediction accuracy, Root mean square error (*RMSE*) and Mean absolute error (*MAE*) are used.

22 $$\begin{aligned} RMSE(y,y')&=\sqrt{\frac{1}{m}\sum _{i=1}^{m}(y(i)-y'(i))^2} \end{aligned}$$

23 $$\begin{aligned} MAE(y,y')&=\frac{1}{m}\sum _{i=1}^{n}|y(i)-y'(i)| \end{aligned}$$

where *y* and $y'$ refer to the actual and the predicted value, respectively, and *m* is the total number of points. Furthermore, difference between actual data and predicted data is plotted to visualize the clear difference.

### Results analysis

Six batches of fermentation data are selected randomly and used for training of model, and the initial fermentation process model is constructed by offline training. To ensure effectiveness, Leave-one-out (LOO), a cross-validation method is used to achieve the best values of model parameters before the testing process. Then two batches fermentation data from remaining four batches are selected randomly and used to correct the model (cross-validation test) offline. Finally, hybrid ICS-MLSSVM is designed by using the best optimum model parameters of MLSSVM obtained through the training and cross-validation phase. The last two batches are used to estimate the key biochemical variables (‘*X*’ cell concentration, ‘*S*’ substrate concentration, ‘*P*’ product concentration) of l-lysine fermentation to verify the online identification accuracy of the model. To find the optimum parameter values of MLSSVM, the range for selection of parameters is defined as $\gamma \in [2^{-5},2^{-3},\dots ,2^{15}]$, $\lambda \in [2^{-10},2^{-8},\dots ,2^{10}]$, $\sigma \in [2^{-15},2^{-13},\dots ,2^{3}]$. The parameters of ICS optimization algorithm are set to as $N_{max}=150$, dimension $dim=3$ (number of MLSSVM parameters to be optimized), $p_{amax}=0.5$, $p_{amin}=0.5$, $\alpha _{max}=0.5$, $\alpha _{min}=0.01$, $\mu =1.5$ and population size is set to $n=24$. These optimal parameter values that produced best results are selected after extensive simulations and employing different combinations. For example, different population size values are applied in range $[10,\dots ,50]$. At $n=24$, we got best results and further increase in value has no significant improvement in results. Initially, $p_{amax}$ is adjusted to 0.9 and different values are used in range $[0.1,\dots ,0.9]$. $p_{amin}$ value is selected in range $[0.01,\dots ,0.5]$ such that $p_{amin}<p_{amax}$. As step size is fixed in standard CS, this work employs adaptive strategy to use variable step size. The values of $\alpha _{max}$ and $\alpha _{min}$ are selected after employing different values in range $[0.01,\dots ,1]$ such that $\alpha _{max}>\alpha _{min}$. Default value of $\mu$ in standard CS provides best performance in ICS. The data is normalized within the range [0, 1] before training.

**Figure 3:**
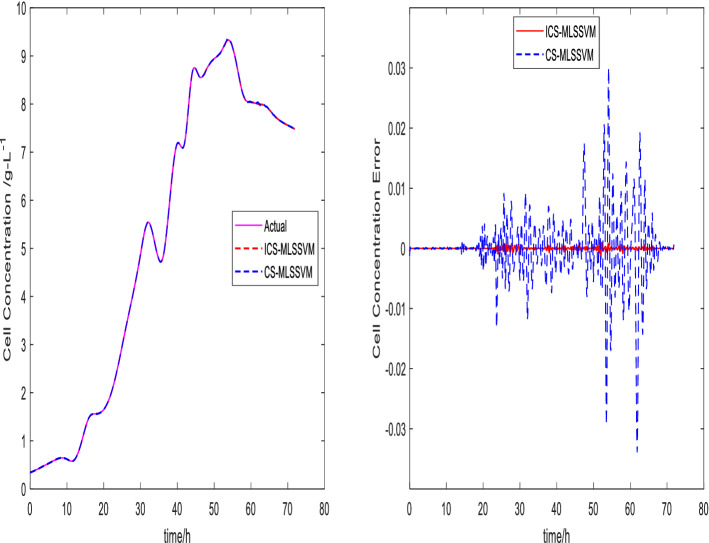
Prediction and error curves of ICS and CS based multi-output MLSSVM (cell concentration).

**Figure 4:**
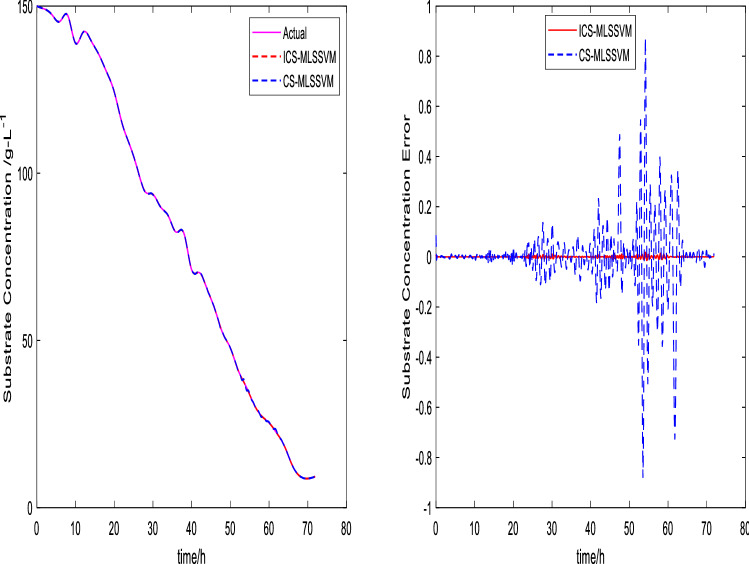
Prediction and error curves of ICS and CS based multi-output MLSSVM (substrate concentration).

**Figure 5:**
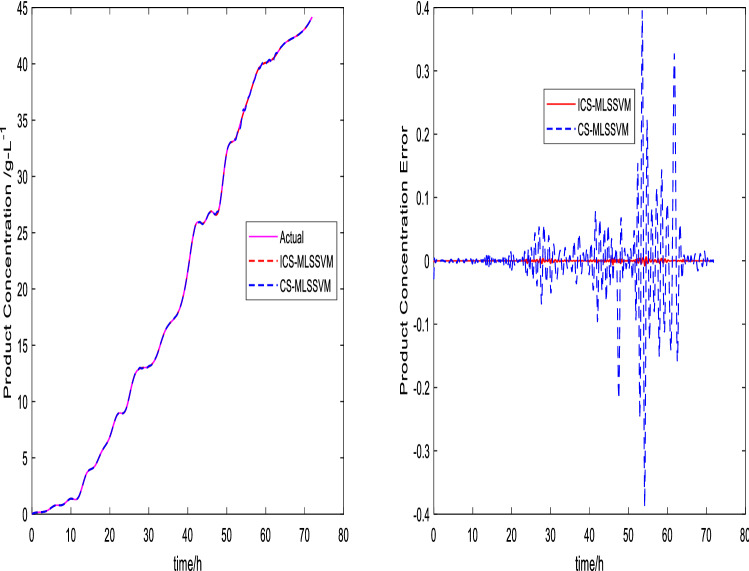
Prediction and error curves of ICS and CS based multi-output MLSSVM (product concentration).

**Figure 6:**
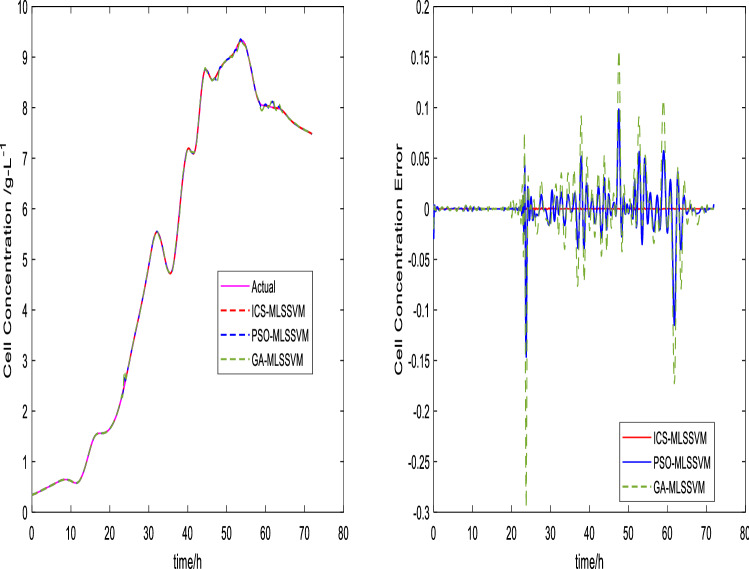
Prediction and error curves of ICS, PSO and GA based multi-output MLSSVM (Cell Concentration).

**Figure 7:**
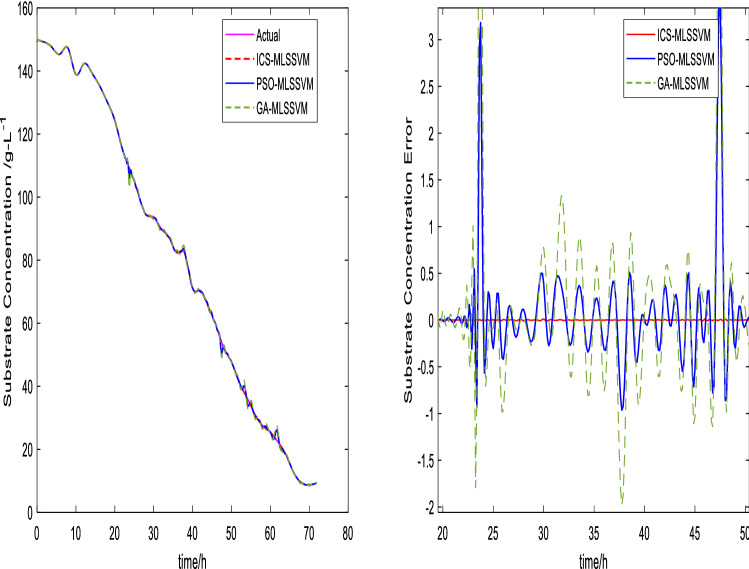
Prediction and error curves of ICS, PSO and GA based multi-output MLSSVM (substrate concentration).

**Figure 8:**
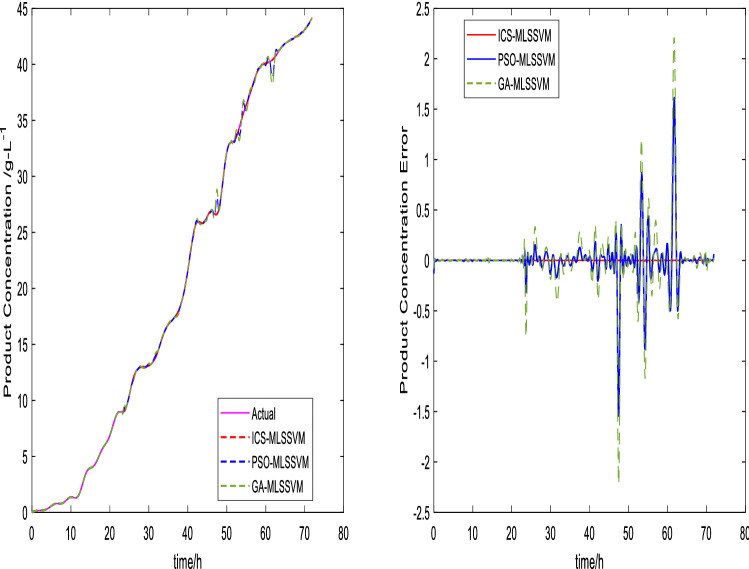
Prediction and error curves of ICS, PSO and GA based multi-output MLSSVM (product concentration).

The prediction curves are shown in Figs. 3, 4, 5, 6, 7 and 8. ICS-MLSSVM is compared with standard CS to show the improved performance of ICS in Figs. 3, 4 and 5. Furthermore, the proposed method ICS-MLSSVM is compared with the MLSSVM based on well known optimization algorithms PSO (PSO-MLSSVM) and GA (GA-MLSSVM) to verify the effectiveness of ICS optimization algorithm in Figs. 6, 7 and 8. As it can be seen from these Figures that ICS-MLSSVM results are much closer to actual value than the CS-MLSSVM, PSO-MLSSVM and GA-MLSSVM.

Furthermore, error plots on right-hand side of Figs. 3, 4, 5, 6, 7 and 8 provide a clear difference between ICS-MLSSVM and corresponding comparitive methods. As the y-axis (output concentrations) is much bigger as compared to error exists between actual and predicted curves, so it is difficult to visualize it. Thus, these error values in plots are determined by calculating difference between actual and predicted values $(y-y')$ to visualize error between actual (*y*) and predicted values $(y')$. A significant difference can be observed in error analysis presented in these plots. All outputs (cell, substrate, product concentration) in such batch fermentation data set are correlated with each other. For example, if the substrate is consumed (concentration decreases), the cell and product concentrations will increase at the same time. However, traditional single output LSSVM can predict only one output at a time, so correlation among all outputs is not considered in the training process which affects the prediction accuracy of the model. The proposed MLSSVM regression model exploits correlation and contrast information among all outputs to find an accurate mapping function between multivariate input space and multivariate output space.

**Figure 9:**
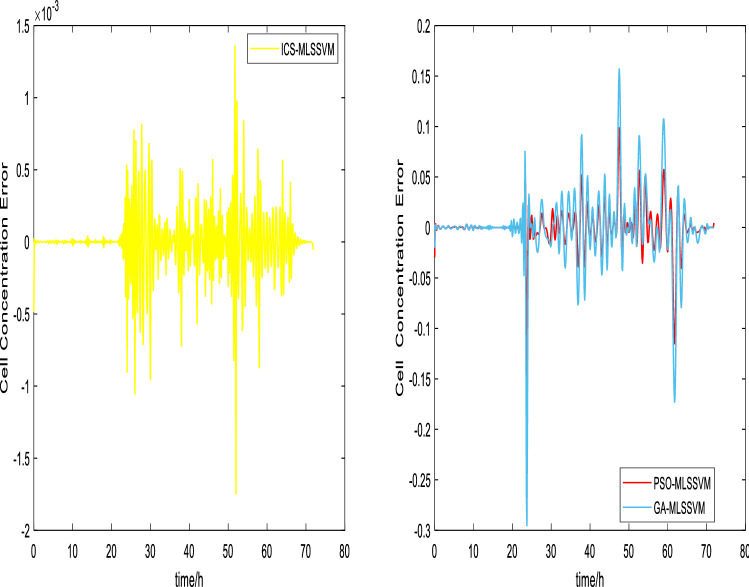
Error curves of ICS, PSO and GA based multi-output MLSSVM (cell concentration).

**Figure 10:**
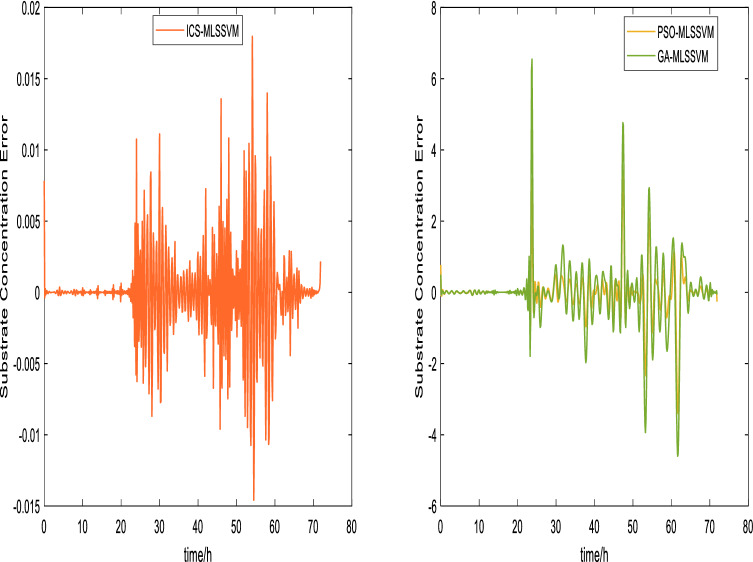
Error curves of ICS, PSO and GA based multi-output MLSSVM (substrate concentration).

**Figure 11:**
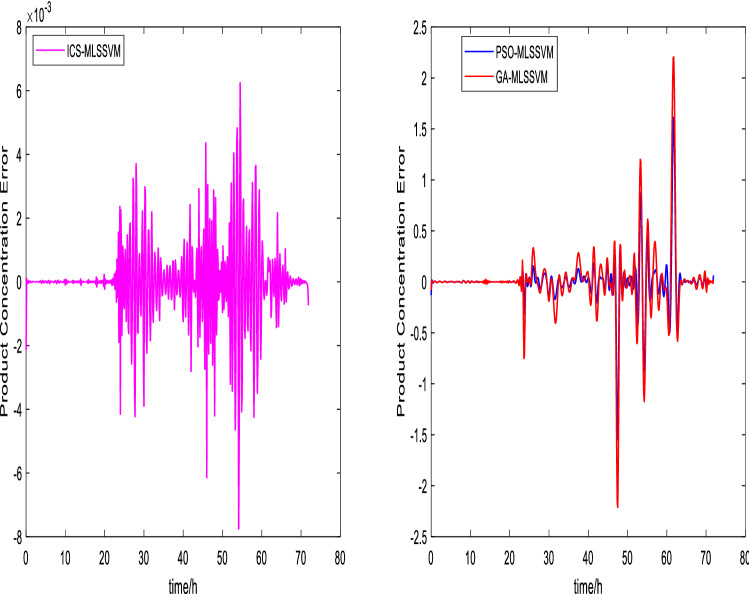
Error curves of ICS, PSO and GA based multi-output MLSSVM (product concentration).

Table 1, illustrates the Root mean square error (*RMSE*) value comparison, and Table 2, compares Mean absolute error (*MAE*). Evaluating both tables reveals the significance of the proposed method for Multi-output regression problems. The *RMSE* and *MAE* values show that a major difference exist between ICS-MLSSVM, CS-MLSSVM, PSO-MLSSVM and GA-MLSSVM. The notable observation after comparing with CS-MLSSVM, PSO-MLSSVM and GA-MLSSVM is that the proposed ICS-MLSSVM method learns the correlation information that exists between outputs and predict all outputs variation trend with negligible error. The reason is that the selection of model parameters of MLSSVM has a very significant part in learning an accurate mapping between inputs and outputs and prediction accuracy. ICS works well in our case to avoid local minimum and successfully finds a global minimum as a solution. However, CS, PSO and GA are not successful in finding a global minimum in a three dimensional unknown search space. To visualize error plots of proposed ICS-MLSSVM clearly, the error curves are plotted separately for ICS-MLSSVM and PSO-MLSSVM , GA-MLSSVM as shown in Figs. 9, 10 and 11. The fitting degree of ICS-MLSSVM is much higher than CS-MLSSVM, PSO-MLSSVM and GA-MLSSVM. It can be seen from these figures that outputs are well predicted by ICS-MLSSVM and magnitude of error spikes is much lower than that of CS-MLSSVM, PSO-MLSSVM and GA-MLSSVM.

**Table 1:** Root Mean Square Error (RMSE) comparison.

MODEL	RMSE-X ($\text {g l}^{-1}$)	RMSE-S($\text {g l}^{-1}$)	RMSE-P(${\text {g l}}^{-1}$)
ICS-MLSSVM	0.00023997	0.00332239	0.00131493
CS-MLSSVM	0.00594054	0.14433584	0.06334809
PSO-MLSSVM	0.02089268	0.57327379	0.22893520
GA-MLSSVM	0.03749970	0.98317813	0.34545799

**Table 2:** Mean absolute error (MAE) comparison

MODEL	MAE-X($\text {g l}^{-1}$)	MAE-S($\text {g l}^{-1}$)	MAE-P(${\text {g l}}^{-1}$)
ICS-MLSSVM	0.00013668	0.00191516	0.00074509
CS-MLSSVM	0.00334472	0.07068850	0.03040805
PSO-MLSSVM	0.01063935	0.26730779	0.08828116
GA-MLSSVM	0.01906414	0.50458959	0.14683407

## Conclusion

In this paper, a hybrid ICS-MLSSVM soft-sensor modeling method is proposed for measuring crucial parameters of the l-lysine fermentation process. According to the characteristics of multi-input and multi-output in the fermentation process, this paper constructs a multi-output MLSSVM model of the fermentation process to measure the key parameters. The proposed MLSSVM method predicts all outputs simultaneously, exploits the useful correlation information among different outputs and designs only a single model for all outputs. In this way, it also decreases computational time as compared to single output LSSVM in which a new model is designed for each output separately because it can only predict a single output independently. Furthermore, considering the importance of the three crucial model parameters (penalty factors $\gamma$, $\lambda$ and kernel width control factor $\sigma$) of the multi-output MLSSVM regression model to the performance of the soft-sensor model, these model parameter values are selected by utilizing a novel ICS optimization algorithm. ICS replace the traditional methods based on experience and trial-error to choose the model parameters and find optimal values of MLSSVM model parameters, which results in a more accurate soft sensor model, and results show the superiority of ICS as compared to CS, PSO and GA. Simulation results show that the prediction accuracy and adaptability of ICS-MLSSVM outperforms the CS-MLSSVM, PSO-MLSSVM and GA-MLSSVM. The model achieves real-time identification effect based on a few input/output data, thus eliminates the need for an exact kinetics model of the fermentation process. In future, this algorithm can be used to solve complex, non-linear, time-varying, and strongly coupled fermentation problems of industry. In our future work, we are interested to extend this work further and use this model in *Model Predictive Control* to control the fermentation process and maintain the desired conditions to increase the yield.

## References

[1] Yokota A, Ikeda M (2017). Amino Acid Fermentation.

[2] Félix FKDC (2019). L-lysine production improvement: A review of the state of the art and patent landscape focusing on strain development and fermentation technologies. Crit. Rev. Biotechnol..

[3] Wang B, Shahzad M, Zhu X, Rehman KU, Uddin S (2020). A non-linear model predictive control based on grey-wolf optimization using least-square support vector machine for product concentration control in l-lysine fermentation. Sensors.

[4] Zhu X, Rehman KU, Wang B, Shahzad M (2020). Modern soft-sensing modeling methods for fermentation processes. Sensors.

[5] Meleiro LADC, Costa ACD, Maciel R (2005). Non-linear multivariable predictive control of an alcoholic fermentation process using functional link networks. Braz. Arch. Biol. Technol..

[6] Foss BA, Johansen TA, Sørensen AV (1995). Nonlinear predictive control using local models-applied to a batch fermentation process. Control Eng. Pract..

[7] Rahman A, Spurgeon SK, Yan X (2012). Estimation and control of non-linear variables in a continuous fermentation process using sliding mode techniques. Trans. Inst. Meas. Control.

[8] Gustavsson R (2018). Development of Soft Sensors for Monitoring and Control of Bioprocesses.

[9] Mears L, Stocks SM, Sin G, Gernaey KV (2017). A review of control strategies for manipulating the feed rate in fed-batch fermentation processes. J. Biotechnol..

[10] Feng, R., Shen, W. & Shao, H. A soft sensor modeling approach using support vector machines. In *Proceedings of the 2003 American Control Conference, 2003* Vol. 5, 3702–3707 (IEEE, 2003). 10.1109/ACC.2003.1240410.

[11] Yuan X, Li L, Wang Y (2019). Nonlinear dynamic soft sensor modeling with supervised long short-term memory network. IEEE Trans. Ind. Inform..

[12] Liu, Y., Zhu, Z. & Zhu, X. Soft sensor modeling for key parameters of marine alkaline protease mp fermentation process. In *2018 Chinese Control And Decision Conference (CCDC)* 6149–6154 (IEEE, 2018). 10.1109/CCDC.2018.8408209.

[13] Gao X-J (2006). Modeling for penicillin fermentation process based on support vector machine. J. Syst. Simul..

[14] Sang, H., Wang, F., He, D., Chang, Y. & Zhang, D. On-line estimation of biomass concentration and specific growth rate in the fermentation process. In *2006 6th World Congress on Intelligent Control and Automation* Vol. 1, 4644–4648 (IEEE, 2006). 10.1109/WCICA.2006.1713262.

[15] Zhu X, Zhu Z (2018). The generalized predictive control of bacteria concentration in marine lysozyme fermentation process. Food Sci. Nutr..

[16] Wang X, Chen J, Liu C, Pan F (2010). Hybrid modeling of penicillin fermentation process based on least square support vector machine. Chem. Eng. Res. Des..

[17] Han Z, Liu Y, Zhao J, Wang W (2012). Real time prediction for converter gas tank levels based on multi-output least square support vector regressor. Control Eng. Pract..

[18] Duch W, Kacprzyk J, Oja E, Zadrozny S (2005). Artificial Neural Networks: Formal Models and Their Applications-ICANN 2005: 15th International Conference, Warsaw, Poland, September 11–15, 2005, Proceedings.

[19] Kocev D, Džeroski S, White MD, Newell GR, Griffioen P (2009). Using single-and multi-target regression trees and ensembles to model a compound index of vegetation condition. Ecol. Model..

[20] Chen G, Yu J (2005). Particle swarm optimization neural network and its application in soft-sensing modeling. Int. Conf. Nat. Comput..

[21] Robles-Rodriguez, C. E., Bideaux, C., Roux, G., Molina-Jouve, C. & Aceves-Lara, C. A. Soft-sensors for lipid fermentation variables based on pso support vector machine (pso-svm). In *Distributed Computing and Artificial Intelligence, 13th International Conference* 175–183 (Springer, New York, 2016). 10.1007/978-3-319-40162-1_19.

[22] Haifeng S, Weiqi Y, Fuli W, Dakuo H (2007). Support vector machines and genetic algorithms for soft-sensing modeling. Int. Symp. Neural Netw..

[23] Yang Q, Gao H, Zhang W (2017). Biomass concentration prediction via an input-weighed model based on artificial neural network and peer-learning cuckoo search. Chemom. Intell. Lab. Syst..

[24] Jiang H, Xu W, Chen Q (2019). Monitoring of cell concentration during saccharomyces cerevisiae culture by a color sensor: Optimization of feature sensor using aco. Sensors.

[25] Wang B, Yu M, Zhu X, Zhu L, Jiang Z (2019). A robust decoupling control method based on artificial bee colony-multiple least squares support vector machine inversion for marine alkaline protease mp fermentation process. IEEE Access.

[26] Zhang Y, Le J, Liao X, Zheng F, Li Y (2019). A novel combination forecasting model for wind power integrating least square support vector machine, deep belief network, singular spectrum analysis and locality-sensitive hashing. Energy.

[27] Luo C (2019). Short-term traffic flow prediction based on least square support vector machine with hybrid optimization algorithm. Neural Process. Lett..

[28] Civicioglu P, Besdok E (2013). A conceptual comparison of the cuckoo-search, particle swarm optimization, differential evolution and artificial bee colony algorithms. Artif. Intell. Rev..

[29] Rajabioun R (2011). Cuckoo optimization algorithm. Appl. Soft Comput..

[30] Marichelvam M (2012). An improved hybrid cuckoo search (IHCS) metaheuristics algorithm for permutation flow shop scheduling problems. Int. J. Bio-Inspired Comput..

[31] Valian E, Mohanna S, Tavakoli S (2011). Improved cuckoo search algorithm for feedforward neural network training. Int. J. Artif. Intell. Appl..

[32] Wolpert DH, Macready WG (1997). No free lunch theorems for optimization. IEEE Trans. Evol. Comput..

[33] Suykens JA, Vandewalle J (1999). Least squares support vector machine classifiers. Neural Process. Lett..

[34] Vapnik V (2013). The Nature of Statistical Learning Theory.

[35] Vapnik VN (1999). An overview of statistical learning theory. IEEE Trans. Neural Netw..

[36] Azimi H, Bonakdari H, Ebtehaj I (2019). Design of radial basis function-based support vector regression in predicting the discharge coefficient of a side weir in a trapezoidal channel. Appl. Water Sci..

[37] Xu S, An X, Qiao X, Zhu L, Li L (2013). Multi-output least-squares support vector regression machines. Pattern Recogn. Lett..

[38] Yang, X.-S. & Deb, S. Cuckoo search via lévy flights. In *2009 World Congress on Nature & Biologically Inspired Computing (NaBIC)* 210–214 (IEEE, 2009). 10.1109/NABIC.2009.5393690.

[39] Viswanathan G, Raposo E, Da Luz M (2008). Lévy flights and superdiffusion in the context of biological encounters and random searches. Phys. Life Rev..

[40] Yang B, Miao J, Fan Z, Long J, Liu X (2018). Modified cuckoo search algorithm for the optimal placement of actuators problem. Appl. Soft Comput..

[41] Mohapatra P, Chakravarty S, Dash PK (2015). An improved cuckoo search based extreme learning machine for medical data classification. Swarm Evol. Comput..

